# Comparison of uncut Roux-en-Y anastomosis and Billroth-II with Braun anastomosis after distal gastrectomy

**DOI:** 10.3389/fsurg.2024.1390876

**Published:** 2024-03-28

**Authors:** Tianxiao Wei, Zhouqiao Wu, Yufan Chen, Yingai Li, Fei Pang, Fei Shan, Ziyu Li, Jiafu Ji

**Affiliations:** Key Laboratory of Carcinogenesis and Translational Research (Ministry of Education), Gastrointestinal Cancer Center, Peking University Cancer Hospital & Institute, Beijing, China

**Keywords:** gastric cancer, gastrectomy, reconstructive surgical procedure, anastomosis, quality of life

## Abstract

**Background:**

This study aimed to compare the clinical outcomes and patient benefits of uncut Roux-en-Y (URY) anastomosis and Billroth-II with Braun (BB) anastomosis after distal gastrectomy.

**Methods:**

We retrospectively reviewed the data of patients who underwent URY or BB anastomosis after distal gastrectomy between March 2015 and December 2017. Clinical characteristics, survival data, postoperative recovery data, and long-term outcomes were recorded and compared between the two groups.

**Results:**

A total of 231 patients were included, with 167 in the URY group and 64 in the BB group. Kaplan–Meier curves for overall survival showed no differences after propensity score matching (*p* = 0.488). Long-term postoperative quality of life evaluation also showed no significant differences. Compared to the BB group, patients in the URY group had a significantly shorter time to start a liquid diet after propensity score matching (67.6 h vs. 46.5 h, *p* = 0.003), and a lower occurrence of bile reflux on follow-up gastroscopy (*p* < 0.001).

**Conclusion:**

The URY anastomosis appears to be a feasible method for digestive tract reconstruction after distal gastrectomy, resulting in less bile reflux and better postoperative recovery. However, there is no significant difference between URY and BB anastomosis in terms of overall survival and long-term quality of life.

## Background

Since 1881, different techniques for gastrointestinal reconstruction have been invented and improved, including Billroth-I, Billroth-II, and Roux-en-Y anastomosis, which are used after distal gastrectomy. The Billroth-I anastomosis is considered to be the most physiologically appropriate, but only if tension-free anastomosis can be achieved between the remnant stomach and the duodenum. The Billroth-II anastomosis is more commonly used when there is a large proportion of the stomach removed, however, patients with the Billroth-II anastomosis suffer from some complications including dumping syndrome, input loop syndrome and bile reflux gastritis (1).

To alleviate the complications of Billroth-II, the Braun anastomosis, which brings together the input and output loops of the jejunum, has been added to the original procedure and has helped to reduce bile reflux to some extent (2). On the other hand, The Roux-en-Y anastomosis prevents bile reflux better than the Billroth-I and Billroth-II anastomoses and does not cause anastomotic tension, but it has its own problems. Studies in the late 1980s reported that more than 30% of patients who underwent the Roux-en-Y anastomosis experienced postoperative abdominal pain, fullness, nausea and even vomiting, which was defined as Roux-Y stasis syndrome (RSS) (3–6). As a solution, Stiegmann et al (7) first proposed the uncut Roux-en-Y (URY) anastomosis, which they believed would not only prevent bile and pancreatic reflux, but would also prevent RSS.

The clinical utility and potential patient benefits of URY and Billroth-II with Braun (BB) anastomosis after distal gastrectomy are still a subject of debate. A meta-analysis published in 2022 revealed that the incidence of reflux gastritis was significantly lower in the URY group than in the BB group, and URY group exhibited a shorter time to first passage of flatus or defecation and a shorter time to first solid diet than the BB group. This study concluded that URY anastomosis is a safe and effective method after distal gastrectomy, and it is better than BB anastomosis in terms of early postoperative recovery and low incidence of reflux gastritis (8).

Although URY anastomosis may have some benefits, evaluations of both procedures remain incomplete and long-term follow-up is lacking. Therefore, this study aimed to compare overall survival, postoperative recovery data, and long-term postoperative quality of life between patients who underwent URY and BB anastomosis after distal gastrectomy.

## Methods

### Study design

This was a real world, single-site, retrospective cohort study of patients who underwent URY or BB anastomosis after distal gastrectomy of gastric cancer. The study utilized a propensity score matching (PSM) method to minimize selection bias. The study population consisted of patients from Peking University Cancer Hospital who underwent an index distal gastric surgery with D2 Lymphadenectomy between March 2015 and December 2017. Patients who underwent multiple organ resections were excluded from the study. This research was approved by the Ethnical Committee of Peking University Cancer Hospital (2018YJZ56).

### Surgical approaches

#### Billroth-II with Braun

The distal stomach was completely transected 5 cm away from the tumor using a 60 mm linear cutter closure, and the specimen was extracted. The jejunum was selected 25 cm distal to the Treitz ligament, and its lateral wall was cut and prepared for anastomosis. The distal greater curvature of the remnant stomach was cut, and the lateral anastomosis was performed by extending the 60 mm linear cutter closure into both arms of the lateral wall of the jejunum and the distal opening of the remnant stomach. The anastomosis was carefully assessed for tension and torsion, and the common opening was closed using a linear cutter closure. The lateral walls of the input loop, which was located 15 cm from the anastomosis, and the output loop, which was located 30 cm from the anastomosis, were cut. The two arms of the 60 mm linear cutter-closer were inserted to perform a lateral Braun anastomosis, and the common opening was closed using the linear cutter-closer. To reinforce the anastomosis and the gastric stump, interrupted sutures of Vecchio suture were applied to the serosal layer and muscular layer.

#### Uncut Roux-en-Y

After completing the BB anastomosis, the proximal intestine of the input loop is closed using a 45 mm bladeless lumpectomy closure (ATS45NK) consisting of six rows of staples, positioned 3 cm proximal to the gastrointestinal anastomosis. In a minority of cases, TA30 linear closure or Vecchio suture closure was used as an alternative.

### Data collection

An internal hospital database was used as the data source to select records that met the eligibility criteria. The database contains information on all admitted patients, including diagnoses, procedures, hospital costs, complications, and other relevant details. Patient demographics, procedural characteristics, type of reconstruction, and clinical characteristics (such as ASA score, diagnosis, surgical time, intraoperative bleeding, etc.) were collected. All data were retrospectively collected by administrative staff at the hospital and recorded in a separate database for data analysis.

#### General health status of preoperative patients, clinical TNM stage and complication classifications

We utilized the Eastern Cooperative Oncology Group Performance Status (ECOG-PS) to assess the preoperative general health status of patients (9). The initial TNM data was recorded based on the 7th edition of the American Joint Committee on Cancer (AJCC) staging system, and the study cohort was restaged according to the definitions of the 8th edition of the AJCC staging system (10, 11). Postoperative complications were graded using the Clavien-Dindo (CD) scoring system (12).

#### Nutrition assessment

The nutritional risk screening score 2002 (NRS2002), body mass index (BMI) and Onodera index were used to assess the patients’ nutritional status (13, 14).

#### Quality of life evaluation

The European Organization for Research and Treatment of Cancer (EORTC) Quality of life questionnaire (QLQ) core QLQ-C30 questionnaire with a gastric cancer-specific clinical assessment questionnaire QLQ-STO22 (Stomach module) was utilized in this study. These questionnaires were self-reported and self-assessed by the patients, and were administered both pre- and post-surgery. The obtained results were scored and normalized based on different dimensions such as functional, symptomatic, economic, and general health status. It should be noted that the Chinese version of the questionnaire has been translated and validated by Chinese scholars in the clinical setting of gastric cancer treatment (15).

#### Gastroscopy

The patient underwent gastroscopy about 1 year after surgery. The amount of residual food and bile reflux were classified according to classifications proposed by Kubo et al. as shown in Table 1 (16).

**Table 1:** Endoscopic classification of residual food and bile reflux.

Grades	Definition
Residual food	
Grade 0	No residual food
Grade 1	A small amount of residual food
Grade 2	A moderate amount of residual food, but possible to observe the entire surface of the remnant stomach with body rolling
Grade 3	A moderate amount of residual food, which hinders observation of the entire surface even with body rolling
Grade 4	A great amount of residual food, for which endoscopic observation is impossible
Bile reflux	
Grade 0	Absence of bile reflux
Grade 1	Presence of bile reflux, when yellowish liquid was observed in the remnant stomach, it was regarded as bile.

### Statistical analysis

All statistical analyses were performed using R Studio (version 4.2.2) and GraphPad Prism 9. Continuous variables were presented as mean ± standard deviation and tested with the independent t-test between groups if normally distributed, otherwise they were presented as median and interquartile range (IQR) and tested with the Mann–Whitney *U* test. Categorical variables were presented as frequency and percentage and tested with the chi-square test, Fisher's exact test, or Mann–Whitney *U* test. PSM was conducted using the R Studio and MatchIt package. The two groups of patients were matched 1:1 without putting back according to the nearest neighbor matching method with a caliper value of 0.02. Kaplan–Meier curves were used to compare differences in overall survival (OS) between the groups, and statistical significance was assessed using the log-rank test. All tests were bilateral, and *p* < 0.05 was considered statistically significant.

## Results

### Patient characteristics

A total of 231 patients were included in this study, with 167 patients in the URY group and 64 patients in the BB group. A significant difference was observed between the two groups in terms of neoadjuvant chemotherapy (*p* = 0.004) and surgery approach (*p* < 0.001). Other demographic and clinicopathologic characteristics including sex, age, BMI, ASA score, gastrointestinal bleeding, ileus, ECOG-PS, NRS2022 score, postoperative pathology AJCC stage, abdominal surgery history, Onodera index were comparable between the two groups. The detailed demographic and clinicopathologic characteristics of the two groups are presented in Table 2.

**Table 2:** Baseline characteristics of the overall cohort.

Variables	BB (*N* = 64)	URY (*N* = 167)	*p* value
Sex (*n*, %)			0.163
Female	50 (78.1%)	115 (68.9%)	
Male	14 (21.9%)	52 (31.1%)	
Age (mean ± SD)	56.7 ± 9.3	57.1 ± 11.3	0.779
BMI (*n*, %)			0.206
Underweight (<18.5 kg/m^2^)	3 (4.7%)	11 (6.6%)	
Normal weight (≥18.5 kg/m^2^ & <24 kg/m^2^)	28 (43.8%)	88 (52.7%)	
Overweight (≥24 kg/m^2^ & <28 kg/m^2^)	28 (43.8%)	53 (31.7%)	
Obese (>28 kg/m^2^)	5 (7.8%)	15 (9%)	
ASA score (*n*, %)			0.19
1	5 (7.8%)	13 (7.8%)	
2	58 (90.6%)	139 (83.2%)	
3	1 (1.6%)	15 (9%)	
Gastrointestinal bleeding (*n*, %)			0.403
No	55 (85.9%)	150 (89.8%)	
Yes	9 (14.1%)	17 (10.2%)	
Ileus (*n*, %)			0.763
No	61 (95.3%)	156 (93.4%)	
Yes	3 (4.7%)	11 (6.6%)	
ECOG-PS (*n*, %)			0.912
0	47 (73.4%)	124 (74.3%)	
1	16 (25%)	40 (24%)	
2	1 (1.6%)	2 (1.2%)	
3	0 (0%)	1 (0.6%)	
NRS2002 score (*n*, %)			0.258
1	48 (75%)	106 (63.5%)	
2	3 (4.7%)	32 (19.2%)	
3	7 (10.9%)	18 (10.8%)	
4	5 (7.8%)	11 (6.6%)	
5	1 (1.6%)	0 (0%)	
Postoperative pathology AJCC stage (*n*, %)			0.625
0	1 (1.6%)	5 (3%)	
1	27 (42.2%)	69 (41.3%)	
2	15 (23.4%)	48 (28.7%)	
3	20 (31.2%)	41 (24.6%)	
4	1 (1.6%)	4 (2.4%)	
Abdominal surgery history (*n*, %)			0.067
No	59 (92.2%)	138 (82.6%)	
Yes	5 (7.8%)	29 (17.4%)	
Neoadjuvant chemotherapy (*n*, %)			0.004
No	50 (78.1%)	96 (57.5%)	
Yes	14 (21.9%)	71 (42.5%)	
Surgery approach (*n*, %)			<0.001
Laparoscopy-assisted	13 (20.3%)	34 (20.4%)	
Total laparoscopy	39 (60.9%)	54 (32.3%)	
Open surgery	11 (17.2%)	78 (46.7%)	
Conversion laparotomy	1 (1.6%)	1 (0.6%)	
Onodera index [median (IQR)]	50.8 (47.2 to 54.2)	51.6 (47.8 to 55.2)	0.324

BMI, body mass index; ECOG-PS, eastern cooperative oncology group performance status; NRS, nutritional risk screening score; AJCC, American joint committee on cancer; SD, standard deviation; IQR, interquartile range.

### PSM model construction

Examination of the baseline characteristics revealed a significant difference in neoadjuvant chemotherapy and surgery approach between the groups. PSM was used to minimize the impact of latent selective bias. The final matching variables included neoadjuvant chemotherapy, surgery approach, abdominal surgery history, sex, and postoperative pathology AJCC stage. After PSM, a total of 108 patients were successfully matched, with 54 in each group, and no statistically significant differences were observed in the baseline characteristics between the two groups (all *p* > 0.05) (Table 3).

**Table 3:** Baseline characteristics after PSM.

Variables	BB (*N* = 54)	URY (*N* = 54)	*p* value
Sex (*n*, %)			0.82
Female	41 (75.9%)	42 (77.8%)	
Male	13 (24.1%)	12 (22.2%)	
Age (mean ± SD)	56.7 ± 9.7	56.9 ± 10.7	0.925
BMI (*n*, %)			0.735
Underweight (<18.5 kg/m^2^)	3 (5.6%)	4 (7.4%)	
Normal weight (≥18.5 kg/m^2^ & <24 kg/m^2^)	24 (44.4%)	27 (50%)	
Overweight (≥24 kg/m^2^ & <28 kg/m^2^)	22 (40.7%)	14 (25.9%)	
Obese (>28 kg/m^2^)	5 (9.3%)	9 (16.7%)	
ASA score (*n*, %)			0.17
1	5 (9.3%)	3 (5.6%)	
2	48 (88.9%)	47 (87%)	
3	1 (1.9%)	4 (7.4%)	
Gastrointestinal bleeding (*n*, %)			0.375
No	46 (85.2%)	49 (90.7%)	
Yes	8 (14.8%)	5 (9.3%)	
Ileus (*n*, %)			0.495
No	52 (96.3%)	54 (100%)	
Yes	2 (3.7%)	0 (0%)	
ECOG-PS (*n*, %)			0.879
0	40 (74.1%)	39 (72.2%)	
1	13 (24.1%)	15 (27.8%)	
2	1 (1.9%)	0 (0%)	
NRS2002 score (*n*, %)			1
1	40 (74.1%)	39 (72.2%)	
2	3 (5.6%)	6 (11.1%)	
3	5 (9.3%)	5 (9.3%)	
4	5 (9.3%)	4 (7.4%)	
5	1 (1.9%)	0 (0%)	
Postoperative pathology AJCC stage (*n*, %)			0.83
1	27 (50%)	28 (51.9%)	
2	12 (22.2%)	12 (22.2%)	
3	14 (25.9%)	13 (24.1%)	
4	1 (1.9%)	1 (1.9%)	
Abdominal surgery history (*n*, %)			1
No	49 (90.7%)	49 (90.7%)	
Yes	5 (9.3%)	5 (9.3%)	
Neoadjuvant chemotherapy (*n*, %)			1
No	42 (77.8%)	42 (77.8%)	
Yes	12 (22.2%)	12 (22.2%)	
Surgery approach (*n*, %)			1
Laparoscopy-assisted	12 (22.2%)	12 (22.2%)	
total laparoscopy	31 (57.4%)	31 (57.4%)	
Open surgery	11 (20.4%)	11 (20.4%)	
Onodera index (mean ± SD)	50.2 ± 5.8	51.2 ± 6.3	0.397

BMI, body mass index; ECOG-PS, eastern cooperative oncology group performance status; NRS, nutritional risk screening score; AJCC, American joint committee on cancer; SD, standard deviation; IQR, interquartile range.

### Postoperative recovery data after PSM

Compared to the BB group, the URY group had significantly shorter time to start a liquid diet (median (IQR), 46.5 (22.1–68.7) hours vs. 67.6 (43.8–90.7) hours, *p* = 0.003), and shorter operative time (median (IQR), 213.0 (183.0–235.0) minutes vs. 227.0 (197.0–255.0) minutes, *p* = 0.036). There were no significant differences between the two groups in intraoperative bleeding, No. of lymph nodes cleared, the first time to passage of flatus, time to pull drainage, postoperative hospitalization time, or surgical complications (Table 4).

**Table 4:** Comparison of perioperative recovery data between the BB group and URY group.

Variables	BB (*N* = 54)	URY (*N* = 54)	*p* value
Operative time [median (IQR), minutes]	227.0 (197.0–255.0)	213.0 (183.0–235.0)	0.036
Intraoperative bleeding [median (IQR), ml]	80.0 (50.0–100.0)	80.0 (53.0–150.0)	0.198
No. of lymph nodes cleared [median (IQR)]	25.5 (20.0–31.0)	27.0 (20.0–36.0)	0.359
The first time to passage of flatus [median (IQR), hours]	78.3 (66.2–96.9)	71.8 (61.6–96.2)	0.231
The first time on liquid diet [median (IQR), hours]	67.6 (43.8–90.7)	46.5 (22.1–68.7)	0.003
Time to pull drainage [median (IQR), days]	6.0 (5.0–7.0)	5.0 (5.0–6.0)	0.092
Postoperative hospitalization time [median (IQR), days]	11.5 (7.0–13.0)	10.0 (7.0–13.0)	0.183
Surgical complications (*n*, %)			0.063
CD grade 0	48 (88.9%)	47 (87%)	
CD grade 2	6 (11.1%)	6 (11.1%)	
CD grade 4a	0 (0%)	1 (1.9%)	

CD, Clavien-Dindo; SD, standard deviation; IQR, interquartile range.

### Overall survival

**Figure 1:**
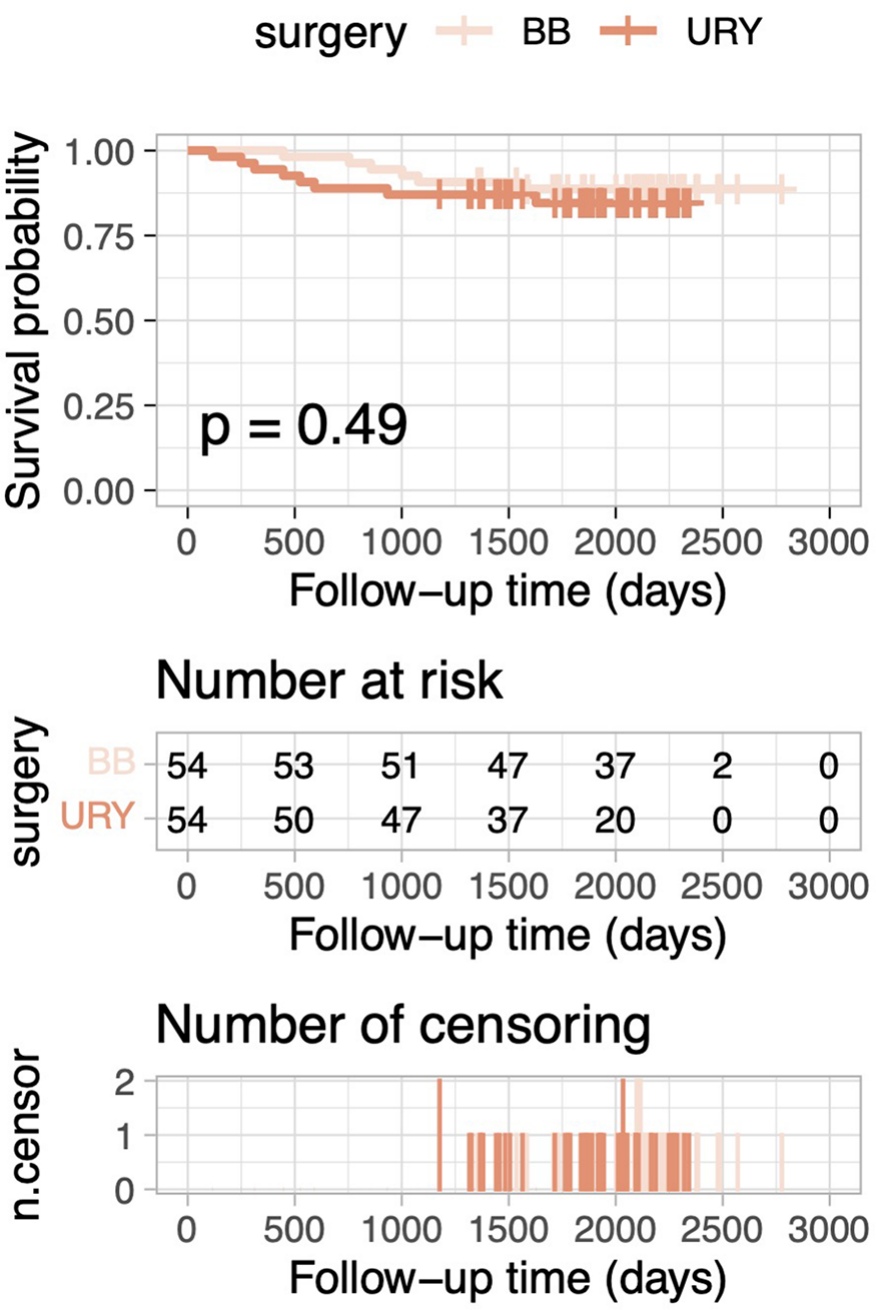
Comparison of overall survival between the URY group and BB group after PSM.

**Figure 2:**
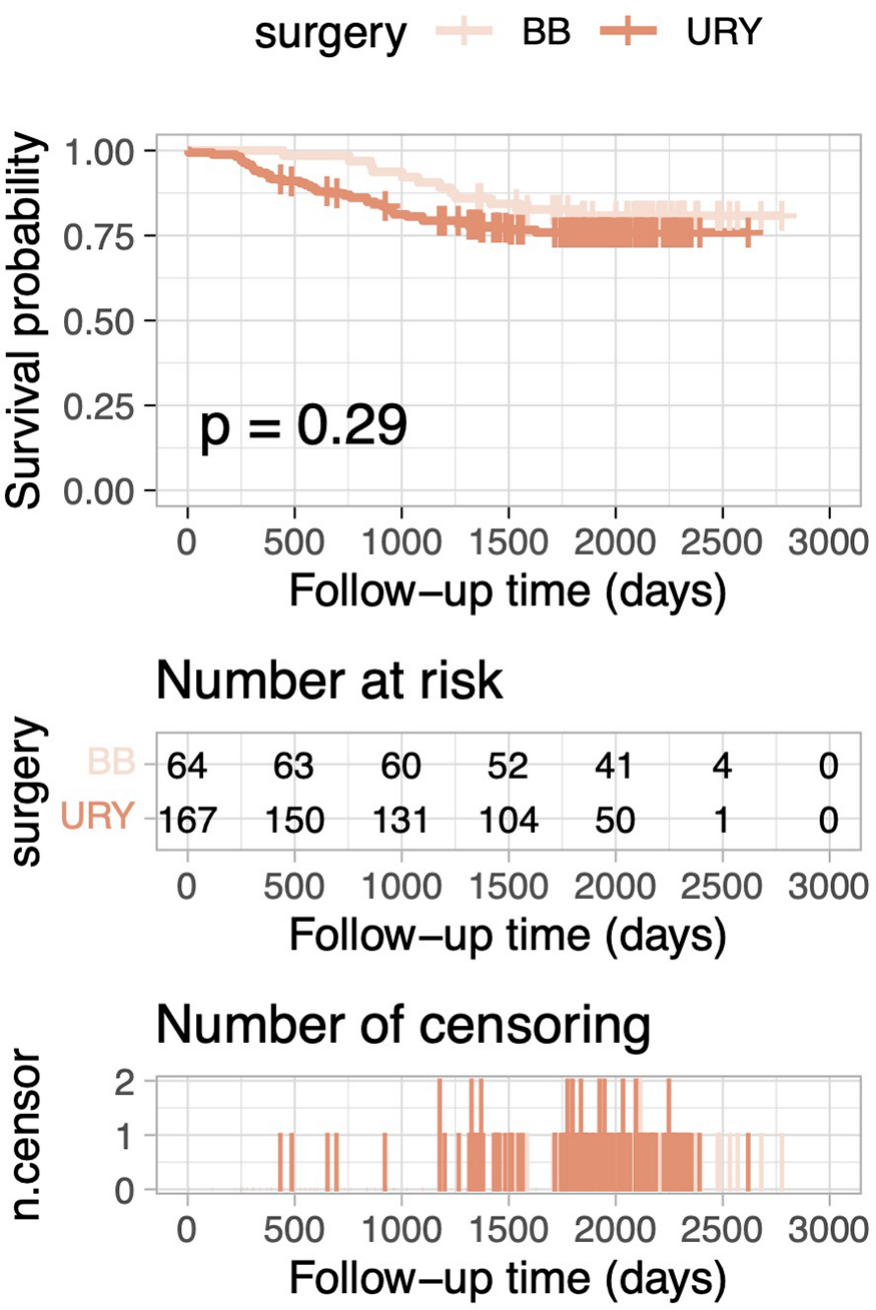
Comparison of overall survival between the URY group and BB group for the overall cohort.

After PSM, the 5-year OS rate was 84.6% in the URY group and 88.8% in the BB group. Kaplan–Meier curves for OS demonstrated no statistically significant difference between the two groups (log-rank test, p = 0.488; Figure 1). Similarly, for the overall cohort before PSM, Kaplan–Meier curves for OS also indicated no statistically significant differences (log-rank test, *p* = 0.287; Figure 2).

### Long-term postoperative quality of life evaluation between the BB group and the URY group

Of all the 231 patients in the overall cohort, 100 patients were followed up after 6 months post-surgery, consisting of 77 patients in the URY group and 23 patients in the BB group. The median follow-up time was 710 days. No statistically significant differences were observed between the two groups (Table 5). The EORTC QLQ-C30 comprises of 30 items including five function scales (physical, role, cognitive, emotional, social), three symptom scales (fatigue, pain, nausea and vomiting), a global health status and QOL scale, and single items (dyspnea, insomnia, appetite loss, constipation, diarrhea, financial difficulties). Each scale or item is transformed into a score ranging from 0 to 100, with higher scores indicating better global health status or functional status, or worse symptom status. The EORTC QLQ-STO22 consists of 22 items, including five scales (dysphagia, chest and abdominal pain, reflux, eating restrictions, anxieties) and four individual items (dry mouth, body image, taste problems, hair loss) that reflecting disease symptoms, treatment side effects, and emotional issues specific to gastric cancer. Higher scores on this questionnaire indicate greater symptomatic problems (17, 18).

**Table 5:** Long-term postoperative quality of life evaluation.

Variables	BB(*N* = 23)	URY (*N* = 77)	*p* value
EORTC QLQ-C30			
Physical function[median (IQR)]	100.0 (93.3–100.0)	93.3 (86.7–100.0)	0.249
Role function[median (IQR)]	100.0 (100.0–100.0)	100.0 (100.0–100.0)	0.756
Emotional function[median (IQR)]	91.7 (83.3–100.0)	100.0 (83.3–100.0)	0.317
Cognitive function[median(IQR)]	100.0 (83.3–100.0)	100.0 (83.3–100.0)	0.358
social function[median(IQR)]	100.0 (75.0–100.0)	100.0 (83.3–100.0)	0.749
Global health status [median (IQR)]	83.3 (70.8–100.0)	83.3 (66.7–100.0)	0.499
Fatigue[median (IQR)]	11.1 (0.0–33.3)	11.1 (0.0–33.3)	0.756
Nausea and vomiting [median (IQR)]	0.0 (0.0–0.0)	0.0 (0.0–0.0)	0.86
Pain [median (IQR)]	0.0 (0.0–0.0)	0.0 (0.0–16.7)	0.275
Dyspnea [median (IQR)]	0.0 (0.0 to 0.0)	0.0 (0.0–0.0)	0.306
Insomnia [median (IQR)]	0.0 (0.0–33.3)	0.0 (0.0–33.3)	0.544
Appetite loss [median (IQR)]	0.0 (0.0 to 33.3)	0.0 (0.0 to 0.0)	0.092
Constipation [median (IQR)]	0.0 (0.0–0.0)	0.0 (0.0–33.3)	0.322
Diarrhea [median (IQR)]	0.0 (0.0–0.0)	0.0 (0.0–33.3)	0.258
Financial difficulties [median (IQR)]	0.0 (0.0–33.3)	0.0 (0.0–33.3)	0.917
EORTC QLQ-STO22			
Dysphagia [median (IQR)]	0.0 (0.0–0.0)	0.0 (0.0–0.0)	0.621
Pain [median (IQR)]	8.3 (0.0–12.5)	8.3 (0.0–16.7)	0.42
Reflux [median (IQR)]	0.0 (0.0–11.1)	0.0 (0.0–11.1)	0.966
Eating [median (IQR)]	0.0 (0.0–8.3)	0.0 (0.0–8.3)	0.667
Anxiety [median (IQR)]	22.2 (0.0–27.8)	11.1 (0.0–22.2)	0.303
Dry mouth [median (IQR)]	0.0 (0.0–33.3)	0.0 (0.0–33.3)	0.317
Taste [median (IQR)]	0.0 (0.0–0.0)	0.0 (0.0–0.0)	0.371
Body image [median (IQR)]	0.0 (0.0–0.0)	0.0 (0.0–0.0)	0.862
Hair loss [median (IQR)]	0.0 (0.0–0.0)	0.0 (0.0–0.0)	0.994

EORTC, European organization for research and treatment of cancer; QLQ, quality of life questionnaire; IQR, interquartile range.

### Gastroscopy results

Of all the 231 patients, 74 underwent gastroscopy approximately a year post-surgery, with 40 in the URY group and 34 in the BB group. The median time of follow-up was 12 months. Comparing the two groups, the URY group had a significantly lower incidence of bile reflux (*p* < 0.001), while there was no significant difference between the groups in terms of residual food. (Table 6).

**Table 6:** Comparison of gastroscopy results between two groups.

Grades	BB (*n* = 34)	URY(*n* = 40)	*p* value
Residual food			0.412
Grade 0	20 (58.8%)	20 (50.0%)	
Grade 1	2 (5.9%)	6 (15.0%)	
Grade 2	0	2 (5.0%)	
Grade 3	7 (20.6%)	5 (12.5%)	
Grade 4	4 (11.8%)	5 (12.5%)	
Unable to judge	1 (2.9%)	2 (5.0%)	
Bile reflux			<0.001
Grade 0	9 (26.5%)	28 (70.0%)	
Grade 1	15 (44.1%)	4 (10.0%)	
Unable to judge	10 (29.4%)	8 (20.0%)	

## Discussion

Currently, various techniques are available for reconstructing the digestive tract after distal gastrectomy, including Billroth-I, Billroth-II, Billroth-II with Braun, Roux-en-Y, and uncut Roux-en-Y. Nevertheless, the choice of reconstruction methods after distal gastrectomy is still controversial. Surgeons continue to search for the most effective reconstruction method, with the aim of minimizing postoperative complications and ensuring better postoperative quality of life.

Several studies have compared these reconstructing methods. Li et al. conducted a systematic review which revealed that URY anastomosis significantly reduce the rate of reflux gastritis after distal gastrectomy among Billroth-I, Billroth-II, Billroth-II with Braun, and Roux-en-Y anastomosis. Besides, URY tended to be a more favorable method due to its operative simplicity, safety, and some other reasons (19). A meta-analysis compared laparoscopic URY and BB anastomosis after distal gastrectomy which found that URY anastomosis is better than BB in terms of early postoperative recovery and low incidence of reflux gastritis (8). Wang et al. reported that none of the patients in the URY group experienced bile reflux on gastroscopy or upper gastrointestinal contrast examination at 3 months post-surgery, whereas 29.03% of patients in the BB group experienced bile reflux (*p* < 0.0001). And the bile reflux at 6 months after surgery was also significantly more frequent in the BB group than in the URY group (20). In the present study, we collected the gastroscopy results of the overall cohort, with 74 patients undergoing this examination approximately one year after surgery. The results demonstrated significantly less bile reflux occurring in the URY group than in the BB group (*p* < 0.001), which is consistent with findings from previous literature. Regarding the postoperative recovery data, the first time to start a liquid diet in URY group patients was significantly earlier than that in BB group patients (*p* = 0.001), indicating that the gastrointestinal function of URY group patients may recover faster and meet the clinical criteria for liquid diet earlier, which is similar with previous studies. Additionally, other postoperative data, such as intraoperative bleeding and surgical complications, did not differ significantly between the URY and BB groups, which may suggest that both procedures are equally safe. Furthermore, studies have shown that the Biofragmentable Anastomotic Ring (BAR) is a safe and time-efficient method for performing Roux-en-Y jejunojejunostomy in gastric cancer surgery (21). Exploring different anastomotic techniques, such as BAR, may be a new way to improve the safety and quality of gastric cancer surgery.

To our knowledge, our study is the first to report on the comparison of OS between the URY and BB groups following distal gastrectomy. The results showed no significant difference between these two groups. Additionally, our study was unique in its reporting of long-term postoperative quality of life. We found no significant differences between the URY group and BB group in any of the measured variables, including reflux symptoms. This may be due to several reasons. Firstly, bile reflux in the remnant stomach does not necessarily correlate with reflux symptoms in patients who have undergone distal gastrectomy, as the cardia structure remains intact and should theoretically provide a normal anti-reflux effect. Secondly, the sample size was relatively small and the follow-up data for the post-operative quality of life questionnaire was lost to a greater extent. Furthermore, we used the BB anastomosis for comparison, which adds a Braun anastomosis to the traditional Billroth-II procedure, allowing bile and pancreatic juice to be diverted from the proximal to the distal jejunum through the Braun anastomosis, thus reducing the amount of refluxed bile (2). Some studies suggest a possible association between bile reflux and gastric stump cancer or reflux symptom, but no difference shown in our study between URY group and BB group (22, 23).

In our study, there were several significant differences observed between the two groups before PSM. A higher percentage of patients received neoadjuvant chemotherapy in the BB group than URY group, and more total laparoscopy approach was used in the BB group while more open surgeries in the URY group. The above factors may affect the outcomes of the surgery. Therefore, in the PSM analysis, included neoadjuvant chemotherapy, surgery approach, abdominal surgery history, sex, and postoperative pathology AJCC stage as matching variables. After 1:1pairing, no statistically significant differences were observed between the two groups, which enhanced the comparability of the study groups. For the long-term postoperative quality of life evaluation and gastroscopy follow-up results, the data were not sufficient to utilize the PSM model. The collection of follow-up data was conducted during patients’ post-discharge outpatient visits at our hospital. However, as many of our patients came from different provinces, they received post-discharge treatment in hospitals within their own provinces. Therefore, comprehensive data on long-term postoperative quality of life and gastroscopy follow-up were not available for analysis using PSM model.

With respect to postoperative complications, the total incidence of complications was 14.72% (34/231). No significant difference was found in postoperative complications between the URY group and the BB group after PSM. Among the 167 URY patients, 16 cases (9.6%) of CD grade 2 or lower complications, 6 cases (3.6%) of CD grade 3–4 complications, and 1 case (0.6%) of perioperative death due to myocardial infarction were observed. There was 1 case (0.6%) of anastomotic leakage related to the anastomotic site and 1 case (0.6%) of diarrhea. Wang et al (20) conducted a randomized controlled trail (RCT) study comparing the postoperative complications between the URY group and the BB group, which showed no significant difference (URY vs. BB, 4.84% vs. 6.45%, *p* = 0.70). Uyama et al (24) retrospectively analyzed 42 patients who underwent laparoscopy-assisted URY anastomosis and reported an overall postoperative complications rate of 4.8%. Yang et al (25) reported an overall postoperative complications rate of 7.6% in 79 cases of laparoscopy-assisted URY anastomosis in an RCT study. A multicenter prospective cohort study conducted in China reported an overall postoperative complication rate of 18.14% (412/2271) for gastric surgeries (26). Our study’s results were comparable to the former studies. Although the postoperative complication rate in our study was marginally higher than some studies, this discrepancy could potentially be attributed to differences in the recognition and registration of postoperative complications of gastric cancer in different centers.

The limitations of this study included: firstly, the sample size of BB group was relatively small. Although PSM was utilized to reduce bias, it was not possible to completely eliminate all confounding factors. Secondly, the scarcity of follow-up data on quality of life may have masked some of the actual differences between the two groups. Thirdly, short-term quality of life data was not collected due to the initial oversight regarding the significance of patients’ quality of life in the early years.

## Conclusion

The URY anastomosis appears to be a feasible method for digestive tract reconstruction after distal gastrectomy, resulting in less bile reflux and better postoperative recovery. However, there is no significant difference between URY and BB anastomosis in terms of overall survival and long-term quality of life for patients.

## Data Availability

The raw data supporting the conclusions of this article will be made available by the authors, without undue reservation.
